# Patterns of Head and Neck Injuries in Urban India: A Multicenter Study

**DOI:** 10.1177/2473974X221128217

**Published:** 2022-10-11

**Authors:** Eric K. Kim, Deepak Suri, Anshul Mahajan, Prashant Bhandarkar, Monty Khajanchi, Anita Gadgil, Kavitha Ranganathan, Martin Gerdin Warnberg, Nobhojit Roy, Nakul P. Raykar

**Affiliations:** 1 University of California San Francisco, School of Medicine, San Francisco, California, USA; 2 Program in Global Surgery and Social Change, Harvard Medical School, Boston, Massachusetts, USA; 3 Harvard School of Dental Medicine, Boston, Massachusetts, USA; 4 Government Medical College, Amritsar, India; 5 Tata Institute of Social Sciences School of Health Systems Studies, Deonar, Maharashtra, India; 6 Department of Surgery, King Edward Memorial Hospital, Mumbai, Maharashtra, India; 7 World Health Organization Collaborating Centre for Research in Surgical Care Delivery in Low-and-Middle Income Countries, Mumbai, India; 8 Division of Plastic Surgery, Brigham and Women's Hospital, Boston, Massachusetts, USA; 9 Department of Global Public Health, Karolinska Institutet, Stockholm, Sweden; 10 Division of Trauma, Emergency Surgery, Surgical Critical Care, Department of Surgery, Brigham and Women's Hospital, Boston, Massachusetts, USA; 11 Center for Surgery and Public Health, Brigham and Women's Hospital, Boston, Massachusetts, USA

**Keywords:** global surgery, trauma, India, low- and middle-income country, head and neck

## Abstract

**Objective:**

The pattern of head and neck injuries has been well studied in high-income countries, but the data are limited in low- and middle-income countries, which are disproportionately affected by trauma. We examined a prospective multicenter database to describe patterns and outcomes of head and neck injuries in urban India.

**Study Design:**

Retrospective review of trauma registry.

**Setting:**

Four tertiary public hospitals in Mumbai, Delhi, Kolkata.

**Methods:**

We identified patients with isolated head and neck injuries using *International Classification of Diseases, 10th Revision* (*ICD-10*) codes and excluded those with traumatic brain and/or ophthalmic injuries and injuries in other body regions.

**Results:**

Our cohort included 171 patients. Most were males (80.7%) and adults aged 18 to 55 years (60.2%). Falls (36.8%) and road traffic accidents (36.3%) were the 2 predominant mechanisms of injury. Overall, 35.7% required intensive care unit (ICU) admission, and 11.7% died. More than 20% of patients were diagnosed with “unspecified injury of neck.” Those with the diagnosis had a higher ICU admission rate (51.4% vs 31.3%, *P* = .025) and mortality rate (27.0% vs 7.5%, *P* = .001) than those without the diagnosis.

**Conclusion:**

Isolated head and neck injuries are not highly prevalent among Indian trauma patients admitted to urban tertiary hospitals but are associated with high mortality. Over a fifth of patients were diagnosed with “unspecified injury of neck,” which is associated with more severe clinical outcomes. Exactly what this diagnosis entails and encompasses remains unclear.

Globally, injuries claim more lives than HIV/AIDS, tuberculosis, malaria, and maternal conditions combined.^1,2^ Injuries account for more than 4.4 million deaths and have led to 520 million cases of nonfatal injury-related harm.^3^ Nearly half of this mortality occurs in individuals aged between 15 and 44 years during their most economically productive period. Therefore, the financial and social burden of these injuries far exceeds the immediate medical costs.^4^

Injury to the head and neck represents a major cause of morbidity and mortality. Head and neck trauma comprises facial bone fracture, soft tissue injuries of the face and neck, and dentoalveolar injuries,^5^ which can affect speech, vision, and mastication and lead to lifelong disability.^6,7^ Their management can be uniquely complex as it often requires input from a wide variety of surgical disciplines, including maxillofacial surgery, otolaryngology, plastic surgery, and neurosurgery.^8,9^

While head and neck trauma has been well examined in high-income countries (HICs), the patterns and outcomes of head and neck injuries in low- and middle-income countries (LMICs) remain poorly understood. Among them, India accounts for about 20% of all global trauma deaths, and the impact of head and neck trauma is expected to be grave but underreported.^10^ Indian studies of head and neck trauma have been limited in scope, with each focusing on a type of injury,^11^ one specific injury,^12^ or a single institution.^13-15^ Furthermore, specific injuries to the head and neck are often lost within the analysis of complex polytrauma that includes spinal, orthopedic, and brain trauma. A deeper understanding of the head and neck trauma in India will guide future efforts in research, resource management, and education to improve patient care. We aimed to understand the profile of non–traumatic brain injury (TBI) extracranial head and neck injuries in a large, multicenter Indian trauma data set.

## Methods

### Study Design and Setting

We retrospectively analyzed the Towards Improved Trauma Care Outcomes (TITCO) registry, a multicenter trauma registry containing data of trauma patients admitted to 4 public university hospitals in Mumbai, Delhi, and Kolkata from October 1, 2013 to September 30, 2015.^16^ All the methodological details of the TITCO registry, like inclusion and exclusion criteria, record validation, and study population, are published elsewhere.^16^

### Study Population

To identify patients with head and neck injuries, we used *International Classification of Diseases, 10th Revision* (*ICD-10;* Version: 2010)^17^ and selected patients whose codes corresponded to injuries in the “Head & Neck” region according to the *ICD-10* injury mortality diagnosis matrix.^18^ We excluded patients with TBI and ophthalmic injuries, similar to the approach of Sethi et al.^19^

### Variables

The following data were collected: patient demographics, injury characteristics (transfer status, mode of transportation to the hospital, type and mechanism of injury, date of injury, nature of injury, *ICD-10* code), Glasgow Coma Score (GCS), and clinical outcomes (intubation, surgical airway, admission to the intensive care unit [ICU], operative management, and mortality). Missing data were excluded.

### Statistical Methods

We characterized the quantitative data with descriptive statistics (mean, standard deviations, percentages). To compare categorical variables between patients with and without unspecified neck injuries, we used the Student *t* test for normally distributed data and the Wilcoxon rank-sum test for nonnormally distributed data for continuous variables and the χ^2^ test or Fisher exact test for categorical variables. Stata (Version 16.0; StataCorp LLC) was used.

### Ethics Approval

The ethics boards of participating hospitals approved the database and permitted a waiver of informed consent: EC/NP-279/2013 RP-01/2013 (All India Institute of Medical Sciences Ethics Committee), IEC/11/13 (Lokmanya Tilak Municipal Medical College and Lokmanya Tilak Municipal General Hospital institutional Ethics Committee), IEC/279 (Institute of Post Graduate Medical Education and Research [IPGME&R] Research Oversight Committee), and IEC(I)OUT/222/14 (Seth GS Medical College and King Edward Memorial Hospital Institutional Ethics Committee).

## Results

Of the 16,047 patients in the TITCO cohort, we identified 870 patients who sustained head and neck injuries after excluding those with TBI and ophthalmic injuries (5.4% prevalence of head and neck injuries among trauma patients). Among these, 699 (80.3%) were polytrauma patients. In the order of decreasing frequencies, concomitant injuries with head and neck injuries in this group occurred in the following body regions: torso (74.0%), extremities (52.1%), unspecified/unclassifiable by body region (40.6%), and spine and upper trunk (16.2%).

**Figure 1.**
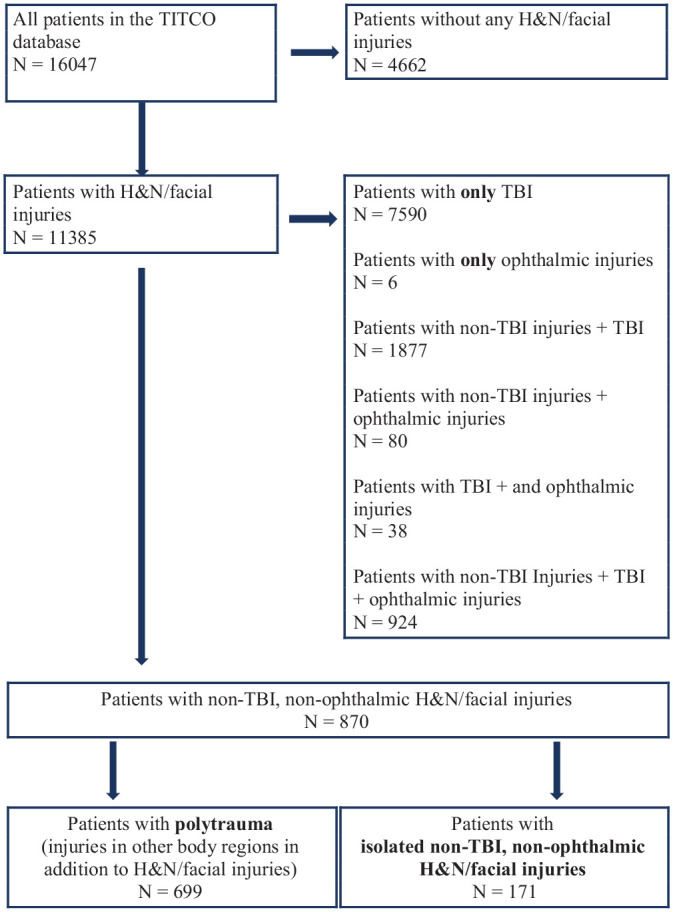
Patient flow diagram. H&N, head and neck; TBI, traumatic brain injury; TITCO: Towards Improved Trauma Care Outcomes.

In total, 171 patients had isolated head and neck injuries (1.1%) among admitted trauma patients. Among these, 37 patients (21.6%) were diagnosed with “unspecified injury of neck” (*ICD-10* code S19.9). Among the 134 patients who were not diagnosed with “unspecified injury of neck,” the most common categories of injury were superficial (51.5%), fracture (18.7%), open wound (14.2%), and burn (10.4%). This is shown in the patient flowchart (**Figure 1**).

### Isolated Head and Neck Injuries (n = 171 Patients)

Demographic and clinical characteristics are shown in **Table 1**. The mean (SD) age of the 171 isolated head and neck patients was 30.6 (19.8) years, with 60.2% of patients aged 18 to 55 years. In total, 80.7% of patients were male, with a male-to-female ratio of 4.2:1. The majority of patients (59.8%) were transferred from other hospitals. The most common mode of transportation was the ambulance (55.7%). Falls and road traffic accidents (RTAs), the 2 top mechanisms of injury, accounted for 36.8% and 36.3% of injuries, respectively. Only 7.6% of injuries were penetrating. For neurologic status, nearly three-quarters (74.8%) of patients had mild GCS; 11.9% and 13.3% had moderate and severe GCS, respectively.

**Table 1.** Patient Demographics of Patients With Isolated Head and Neck Injuries (n = 171).^a^

Characteristic	
Age, mean (SD), y	30.6 (19.8)
Age group, y	
0-17	52 (30.4)
18-55	103 (60.2)
55+	16 (9.4)
Male Sex	
Male	138 (80.7)
Patient transferred from other hospital	
Yes	101 (59.8)
Missing data	2
Mode of transportation to hospital	
Ambulance	93 (55.7)
Police	13 (7.8)
Private car	27 (16.2)
Other (taxi, motor rickshaw)	34 (20.4)
Missing data	4
Mechanism of injury	
Assault	19 (11.1)
Burn	13 (7.6)
Fall	63 (36.8)
Other	14 (8.2)
Road traffic accidents	62 (36.3)
Penetrating injury	13 (7.6)
Glasgow Coma Score	
Mild	107 (74.8)
Moderate	17 (11.9)
Severe	19 (13.3)
Missing	28
Intubation within 24 hours of arrival	22 (12.9)
Surgical airway within 24 hours of arrival	4 (2.3)
ICU admission	61 (35.7)
Obtained CT scan	165 (96.5)
Received operative management within 24 hours of arrival	8 (4.7)
Length of stay for survivors, mean (SD), d (n = 151)	12.2 (32.9)
Length of survival for patients who died, mean (SD), d (n = 20)	11.1 (15.8)
Died	20 (11.7)

Abbreviations: CT, computed tomography; ICU, intensive care unit.

a Values are presented as number (%) unless otherwise indicated.

Diagnostically, 96.5% of the cohort obtained computed tomography (CT) imaging. In the first 24 hours of admission, 2.3% got a surgical airway, 12.9% received intubation, and 4.7% received operative management. In total, 35.7% of patients were admitted to the ICU. The mean length of stay was 12.2 days, and the mean length of survival for those who died was 15.8 days. The overall mortality rate of patients with isolated head and neck injuries was 11.7%.

### Unspecified Injury of Neck (n = 37 Patients)

Patients diagnosed with unspecified neck injury differed in both demographic and clinical profiles from those without the diagnosis (shown in **Table 2**). Those with unspecified neck injuries were on average older than those without the diagnosis (41.2 vs 27.7, *P* = .0002), with a higher proportion of patients in the age group 18 to 55 years (78.4% vs 59.0%, *P* < .001). Mechanisms of injury differed between patients with and without unspecified neck injury (*P* = .0004), with a higher proportion of patients with unspecified neck injuries experiencing falls (56.8% vs 31.3%).

**Table 2.** Demographics and Clinical Outcomes of Patients With vs Without Unspecified Neck Injury.^a^

Characteristic	Patients with unspecified neck injury (n = 37)	Patients without unspecified neck injury (n = 134)	*P* value
Age, mean (SD), y	41.2 (27.7)	27.7 (19.4)	.0002^b^
Age group, y			.001^b^
0-17	3 (8.1)	49 (36.6)	
18-55	29 (78.4)	79 (59.0)	
55+	5 (13.5)	6 (4.5)	
Male sex	34 (91.9)	104 (77.6)	.060
Patient transferred from other hospital			.064
Yes	27 (73.0)	74 (56.1)	
Missing data	0	2	
Mode of transportation to hospital			.104
Ambulance	23 (62.2)	70 (53.9)	
Police	2 (5.4)	11 (8.4)	
Private car	9 (24.3)	18 (13.9)	
Other (taxi, motor rickshaw)	3 (8.1)	31 (23.9)	
With unspecified (n= 27)	0	4	
Mechanism of injury			.004^b^
Assault	1 (2.7)	18 (13.4)	
Burn	0 (0)	13 (9.7)	
Fall	21 (56.8)	42 (31.3)	
Other	5 (13.5)	9 (6.7)	
Road traffic accidents	10 (27.0)	52 (38.8)	
Penetrating injury	1 (2.7)	12 (9.0)	.303
Glasgow Coma Score			.575
Mild	28 (82.3)	79 (72.5)	
Moderate	3 (8.8)	14 (12.8)	
Severe	3 (8.8)	16 (14.7)	
Missing data	3	25	
Intubation within 24 hours of arrival	8 (21.6)	14 (10.5)	.072
Surgical airway within 24 hours of arrival	0 (0)	4 (3.0)	.578
ICU admission	19 (51.4)	42 (31.3)	.025^b^
Obtained CT scan	31 (83.8)	134 (100)	.0001^b^
Received operative management within 24 hours of arrival	1 (2.7)	7 (5.2)	1.000
Length of stay for survivors, mean (SD), d (n = 151)	16.1 (20.1)	11.3 (35.1)	.0056^b^
Length of survival for patients who died, mean (SD), d (n = 20)	16.4 (17.7)	5.7 (12.2)	.0937
Died	10 (27.0)	10 (7.5)	.001^b^

Abbreviations: CT, computed tomography; ICU, intensive care unit.

a Values are presented as number (%) unless otherwise indicated.

b Statistically significant, *P* < .05.

For imaging, the 6 patients who did not get CTs were all diagnosed with an unspecified neck injury. A lower rate of patients with unspecified neck injuries obtained CT scans than those without unspecified neck injuries (83.8% vs 100%, *P* = .0001). Although not statistically significant, patients with unspecified neck injuries were intubated at a higher rate than patients without them (21.6% vs 10.5%, *P* = .072). The rates of operation between patients with unspecified neck injury and those without were 2.7% and 5.2%, respectively (*P* = 1.000). The 8 operations undertaken by those without unspecified neck injury included neck exploration, with 3 receiving additional surgeries (laryngeal repair and jugular vein ligation, n = 1; tracheal repair, n = 1; and cartilage repair, n = 1). The 1 patient with an unspecified neck injury who received surgery underwent “exploration and primary repair.” A higher proportion of patients with unspecified neck injuries were admitted to the ICU (51.4% vs 31.3%, *P* = .025). The mean length of hospital stay was longer for those unspecified neck injuries (16.1 vs 11.3 days, *P* = .0056). The morality rate was higher for patients with unspecified neck injuries (27.0% vs. 7.5%, *P* = .001). The mean length of survival for those who died was also longer for those who sustained unspecified neck injuries, although the difference was statistically nonsignificant (16.4 vs 5.7 days, *P* = .0937).

## Discussion

This multicenter database study found a 1.1% prevalence of isolated head and neck injuries among trauma patients admitted to tertiary hospitals in urban India. More than one-fifth of these patients were diagnosed with an “unspecified injury of the neck.” While the overall mortality of patients with all isolated head and neck injuries was 11.7%, those with unspecified neck injuries had a significantly higher mortality rate of 27%.

Isolated extracranial head and neck injuries account for only a small fraction of trauma admissions in urban India. About 5% of all admitted Indian trauma patients had a head and neck injury, and 1% sustained an isolated head and neck injury. While the reported prevalence varies widely depending on study settings and populations, 1 study found that 27.5% of major trauma patients had a head and neck injury.^20^ There are several potential reasons for these seemingly low figures. First, most head and neck injuries do not warrant hospital admission and can be addressed in urgent or emergency care settings.^19^ Second, because this study excluded patients with TBI, which often co-occurs with otorhinolaryngologic injuries,^21,22^ our numbers are lower than those reported by studies that did not exclude intracranial injuries.^20,22^ Third, the 5% prevalence is likely an underestimate, as data collectors might not have coded every single minor head and neck injury in polytrauma patients with a more severe injury in another body region. Regardless, head and neck injuries alone are not a predominant cause of hospitalization in the Indian context.

Demographic patterns of head and neck injuries in urban India were consistent with global patterns of head and neck trauma. They were unsurprisingly more frequent among males and young individuals. The preponderance of males and young people in head and neck and orofacial trauma is well documented and attributed to traditional behavioral and occupational patterns of these groups that predispose them to injury.^12,13,19,21-23^

Falls and RTAs were the 2 most common mechanisms of head and neck injuries in our cohort. Historically, LMICs have been disproportionately affected by RTAs because of poor road conditions and limited transportation infrastructure.^24^ This disparity is reflected in other studies conducted in LMICs^12,13,21^ as well as ours, which showed that RTAs caused injury in over a third of all patients. While RTAs were the top etiology of injury in other Indian studies,^13,23^ falls were equally as culpable for injury as RTAs in our study. We demonstrate that falls may be a bigger factor than previously thought and can lead to head and neck injuries severe enough to require hospitalization in India.

The mortality rate of patients with isolated head and neck injuries in urban India is higher than what has been reported in the global literature, which ranges from less than 1% to 10%.^13,19,20,25^ The wide range of mortality in the literature is ascribed to differences in eligibility criteria and patient populations, with some studies including intracranial injuries and polytrauma patients. The substantial burden of unspecified neck injury observed in our cohort further demonstrates the variability in inclusion, disease burden, and populations reported in these studies. The mortality rate in our study is higher than that of Singhai et al,^13^ who prospectively examined 200 head and neck trauma patients and found an 8% mortality rate.

This high mortality rate may be attributed to several factors. One explanation is the differences between the trauma systems of LMICs and HICs. People in LMICs with equivalent injuries as those in HICs are more likely to die.^26^ Specifically in India, 1 study reported that the odds of mortality were 58 times and 20 times higher in India for mild to moderate injuries in the head and face anatomic regions, respectively.^27^ Delays in surgical care, limited multidisciplinary coordination in trauma care, and lack of treatment protocols all contribute to the gap in trauma outcomes between HICs and LMICs.^28^ These issues are exacerbated by the shortage of surgeons who specialize in craniofacial trauma in low-resource settings, which likely translates to suboptimal care.^29^ These findings together represent a critical need for trauma system strengthening and workforce development in India to address this disparity in head and neck trauma–related mortality.^28^ Another explanation, as detailed in the previous paragraph, is that other studies had different inclusion and exclusion criteria, which makes direct comparison between our results and others studies’ challenging.

The cohort of these patients with “unspecified injury of neck” requires special mention as they comprised 20% and had distinct demographic and clinical characteristics. This group was on average older and had falls at a higher rate, suggesting that patients aged 40 who fell most commonly sustained this injury. This group was also admitted to the ICU at a higher rate, had a longer mean length of stay for survivors, and suffered a higher mortality rate, indicating that they had sustained more serious injuries and required a higher level of care. This nonspecific diagnosis, which is associated with more adverse outcomes, represents an opportunity for intervention, such as improved triaging, clinical education, and resource allocation.

We theorize that “unspecified injury of neck” was used as a catchall diagnosis given to any patient whose diagnosis was unclear at the time of presentation. Notably, the only 6 patients who did not get CTs in the entire cohort were all diagnosed with an unspecified neck injury, which might have contributed to diagnostic uncertainty. The diagnosis likely included a heterogeneous group of injuries, but a few key findings suggest 2 potential injuries that this broad diagnosis could encompass.

One possibility is laryngotracheal trauma. It is particularly hard to diagnose because patients may be asymptomatic for up to 48 hours and have very subtle signs of injury.^30^ Although not statistically significant, the higher rate of intubation in patients with unspecified neck injuries within the first 24 hours suggests a greater occurrence of airway compromise or respiratory distress, which can be signs of laryngotracheal injury. Another consideration is vascular injury. Because the onset of symptoms also greatly varies, vascular imaging such as CT angiography is essential to diagnosis.^31^ While the TITCO data set does not specify the type of CT each patient receives, even CT angiography has demonstrated variable sensitivity for identifying blunt cerebrovascular injury (66%-100%), depending on radiologist expertise, CT technology, and institution-specific thresholds.^31-36^ Both of these conditions are associated with high mortality.

Considering the high concomitance of TBI with head and neck injuries,^21^ we also wondered whether many patients in this cohort had TBI that might have been missed or not coded. The similar distributions of TBI between those with and without the diagnosis do not explain the differential mortality rates, but we must interpret the GCS data carefully. GCS was missing in 16% of the cohort and recorded only at admission. They do not reflect the neurologic status of patients later in the hospital course, which can be misleading for certain conditions, such as cerebrovascular injuries, that may not exhibit neurologic symptoms until hours or days later.

This study has several limitations. First, our selection for patients with isolated head and neck injuries excluded those with TBI or polytrauma who could have also had head and neck injuries. We limited our investigation to isolated injuries because our objective was to assess the characteristics of patients with primary head and neck injuries; grouping all patients with head and neck injuries who also had widely varying pathologies (eg, abdominal injuries, extremity fractures) would have been impractical. Second, the TITCO database has innate limitations and does not report the specifics of clinical outcomes, such as the extent of injury, CT findings, or types of fracture. Despite these limitations, we believe that our study illuminates important trends and profiles of head and neck injury in India and will help inform the design of future trauma studies in LMICs, which can address the gaps in this study. Third, we do not know the details of each patient’s hospital course as most of our data were recorded within the first 24 hours of admission, which limits our understanding of the context that led to certain outcomes, such as mortality. Future studies should examine the longitudinal clinical courses of head and neck patients and identify specific factors associated with mortality. Last, because the TITCO registry consists of data from 4 urban academic hospitals, we do not capture the burden of head and neck injury in rural areas or community settings. We believe that our findings may be generalizable to other LMICs, which have similarly undergone rapid industrialization and urbanization but are not yet equipped with robust trauma systems.^37^

## Conclusion

Isolated head and neck injuries account for a small percentage of hospitalized trauma patients and primarily affect young male patients who had falls or road traffic accidents. The overall mortality associated with isolated head and neck injuries was high, more than previously reported in similar settings and high-income countries. Over a fifth of these patients were diagnosed with “unspecified injury of neck” and were on average older and had more severe presentations, as evidenced by higher ICU admission and mortality rates. The conditions encompassed in the diagnosis “unspecified injury of neck” remain unclear.

## Author Contributions

**Eric K. Kim**, study conception, study design, data collection, data analysis, manuscript drafting and revision; **Deepak Suri**, study design, data collection, data analysis, manuscript drafting and revision; **Anshul Mahajan**, study design, data analysis, manuscript drafting and revision; **Prashant Bhandarkar**, study design, data collection, data analysis, manuscript drafting and revision; **Monty Khajanchi**, study design, data analysis, manuscript drafting and revision; **Anita Gadgil**, study design, data analysis, manuscript drafting and revision; **Kavitha Ranganathan**, study design, data analysis, manuscript drafting and revision; **Martin Gerdin Warnberg**, study design, data analysis, manuscript drafting and revision; **Nobhojit Roy**, study conception, study design, manuscript drafting and revision; **Nakul P. Raykar**, study conception, study design, data analysis, manuscript drafting and revision.

## Disclosures

**Competing interests:** None.

**Sponsorships:** None.

**Funding source:** The Towards Improved Trauma Care Outcomes (TITCO) registry was supported by grants from the Swedish National Board of Health and Welfare and the Laerdal Foundation for Acute Care Medicine, Norway. These organizations did not influence the study conception, manuscript writing, or any other decisions involved in publishing the study.
